# Current Status of Sarcopenia in the Disabled Elderly of Chinese Communities in Shanghai: Based on the Updated EWGSOP Consensus for Sarcopenia

**DOI:** 10.3389/fmed.2020.552415

**Published:** 2020-11-12

**Authors:** Qing Fang, Guoying Zhu, Jianwei Huang, Shayi Pan, Minyan Fang, Qiuting Li, Qin Yin, Xiaoqing Liu, Qingya Tang, Dongping Huang, Jingmin Liu

**Affiliations:** ^1^Department of Clinical Nutrition, People's Hospital of Shanghai Putuo District, Shanghai, China; ^2^Department of Nursing, People's Hospital of Shanghai Putuo District, Shanghai, China; ^3^Department of Clinical Nutrition, Xinhua Hospital Affiliated to Shanghai Jiaotong University School of Medicine, Shanghai, China; ^4^Division of Sports Science and Physical Education, Research Center of Sports and Health Science, Tsinghua University, Beijing, China

**Keywords:** sarcopenia, disability, elderly, handgrip strength, China

## Abstract

Our study aimed to investigate the prevalence and associated factors of sarcopenia in the disabled elderly in communities in Shanghai, China. A cross-sectional study was conducted in 2018. Five hundred and seventy two participants (≥60 years) were recruited through cluster sampling from Putuo District of Shanghai. Sarcopenia was defined according to the updated consensus of the European Sarcoma Working Group in 2019. The sarcopenia, depression, and nutrition status were assessed by using SARC-F, the Short Version of the Center for Epidemiological Studies Depression Scale (CES-D-10), and the Mini Nutritional Assessment-Short form (MNA-SF), respectively Physical activity was also assessed. Our results showed the prevalence of sarcopenia was 0.5%, but the prevalence of low handgrip strength was 37.2% (male, 5.5%; female, 39.1%). The modified Poisson regression model was used to evaluate the relationship among related variables and low handgrip strength. The risk for low handgrip strength was higher in the physically disabled subjects than in the visually disabled ones (aPR: 1.69, 95% CI: 1.88-2.42). Depressive symptoms (aPR: 1.31, 95% CI: 1.04-1.62) and PASE score (aPR: 0.99, 95% CI: 0.99-1.00) were independently associated with low handgrip strength. In summary, the prevalence of EWGSOP2-defined sarcopenia is low and the prevalence of declined muscle strength is high in the disabled elderly. The elderly participants with a physical disability had a higher prevalence of low hand handgrip strength than those with a visual disability. More studies with a larger sample size and longitudinal follow-up are needed to confirm our findings.

## Introduction

As the population ages and life expectancy increases, the number of diseases and/or syndromes associated with aging increases concurrently. Sarcopenia is a progressive, systemic skeletal muscle disease that may increase the risk of falls, fractures, physical disabilities, and even death. Based on the recent scientific and clinical findings on sarcopenia, the European Sarcoma Working Group (EWGSOP) and the Asia Sarcoma Working Group (AWGS) have updated their consensuses on sarcopenia in 2018 and 2019, respectively (1, 2). Both recognize that muscle strength is more important than muscle mass in predicting adverse outcomes. In addition, EWGSOP2 clearly proposes the “F-A-C-S” process for the assessment of sarcopenia: Find (SARC-questionnaire)- Assess (muscle strength)–Confirm (muscle mass)—Severity (physical performance).

According to the World Disability Report 2010, there are currently more than 1 billion disabled people worldwide, accounting for 15% of the total population. Among these people, 600 million are in Asia. According to the Sixth National Census and the Second National Sampling Survey of Disabled Persons, the total number of disabled people in China was 85.02 million at the end of 2010, accounting for 6% of the total population, and people with physical disabilities make up the largest proportion (29.1%). As the prevalence of disability will increase with age and the age of the population is increasing, the number of disabled people in China will continue to increase. The significant increase in the number of elderly disabled people due to population aging has become an important worldwide issue, and the elderly disabled people, or the “dual vulnerable group,” should be paid more attention.

Few studies about sarcopenia have focused on disabled people. Although it is generally accepted that the body muscle mass will naturally decrease due to reduced physical activity, few studies have investigated the status of muscle quantity and quality in the disabled people. Therefore, this study was to investigate the prevalence of sarcopenia and factors related to sarcopenia in the disabled elderly in a Chinese community, which may provide a scientific basis and data support for the development of targeted interventions.

## Materials and Methods

### Study Design and Sampling

This cross-sectional study was conducted in the Putuo District, Shanghai, China. With the aid of the Federation of the Disabled Person, Putuo District, a questionnaire survey and physical examinations were conducted by trained investigators from April 2018 to June 2018. This study was approved by the Ethics Committee of Putuo People's Hospital, Shanghai (2018-12). Informed consent was obtained from each subject before study.

**Figure 1:**
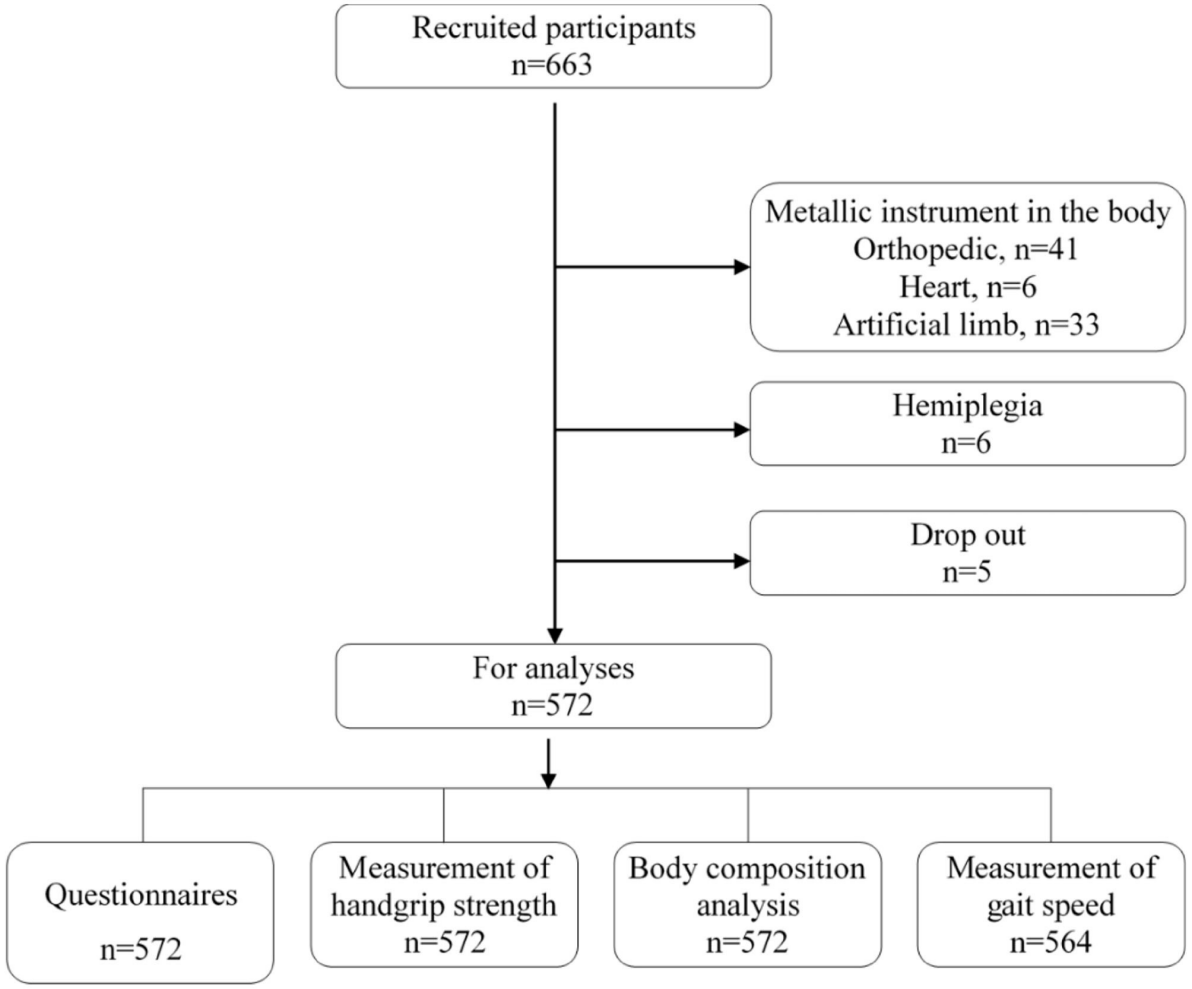
Inclusion diagram for the recruitment of subjects in the present study.

Cluster sampling was employed to recruit disabled people from the communities of the Putuo District, Shanghai, China. The appendicular skeletal muscle mass index (ASMI) was normally distributed with a standard deviation of 0.8 kg/m^2^ as previously reported (3, 4). The admissible error was 0.1 kg/m^2^ and the design effect was 2.0. Considering the response rate was 90%, at least 554 subjects were needed in this study with a power of 0.8 and α error of 0.05. In 2016, there were 31,500 disabled people, of whom about 17,000 disabled people were aged 60 years or older. In addition, there were 10 towns, and subjects were randomly recruited from one town. Finally, a total of 572 subjects were recruited into the present study as shown in Figure 1.

### Subjects

The disabled residents who were aged ≥60 years and could effectively cooperate with investigators were recruited from a community of the Putuo District, Shanghai. The definition, types, and degrees of disability were based on the “Disability Protection Law of People's Republic of China.” Exclusion criteria were as follows: (1) Subjects could not receive body composition measurements and handgrip strength assessments due to physical disability; (2) the disability could affect the bioimpedance analysis (BIA) or the collection of key parameters (such as handgrip strength), due to prosthesis or implantation of metal plates, nails, pacemakers, or stents, or subjects who had hemiplegia due to stroke; (3) subjects had severe cognitive impairment; (4) subjects had acute attacks of chronic disease or stress disease; (5) there was evident edema; and (6) subjects had a poor compliance, and communication between subjects and investigators was difficult. In total, 572 participants were enrolled after the exclusion criteria were applied (Figure 1).

### Observations

The demographic characteristics (age, gender, education level, income, and living status), lifestyle (smoking, drinking, and diet), nutritional risk screening (Mini Nutritional Assessment-Short Form; MNA-SF), chronic diseases, depression (Short Version of Center for Epidemiological Studies Depression Scale [CES-D-10]), history of falls in the past year, risk for sarcopenia (SARC-F questionnaire), and anthropometric data were collected. The Physical Activity Scale for the Elderly (PASE) was used to assess physical activity. The elderly who lived alone or only with their spouse were defined as “empty nest elderly” by asking the subjects for co-residents. Based on the smoking status, subjects were divided into current smokers, ex-smokers, and never smokers, and the current smokers and ex-smokers were defined as smokers. Drinking was defined as use of 25 g of alcohol once or more weekly. At the same time, anthropometric measurements (height, weight, and body composition) and physical performance (handgrip strength, gait speed) assessment were performed.

### Assessments

For the SARC-F questionnaire, the updated EWGSOP consensus and AWGS consensus recommend the use of the SARC-F questionnaire to screen sarcopenia (1, 2). The SARC-F questionnaire assesses five components: strength, assistance walking, rising from a chair, climbing stairs, and falls. The score of each question ranges from 0 to 2, and the total score is 10. A total score equal to or higher than 4 is suggestive of high risk for sarcopenia (5). This scale has been confirmed in the African-American, Longitudinal Baltimore Aging Study, and National Health and Nutrition Survey (6). The reliability and validity of the Chinese version of the SARC-F scale (7) have also been confirmed, and it has been used in the assessment of the elderly in communities and pension institutions in China (8, 9).

Handgrip strength (HS) was measured in kilograms with a hand dynamometer (CSTF-WL, Tsinghua Tongfang, Beijing, China) with subjects in a standing position, and the higher value of two measurements from the dominant hand was used in the following analysis.

For Appendicular Skeletal Muscle Mass (ASM), a multifrequency, segmental, and validated BIA instrument (BCA-2A; Tsinghua Tongfang, Beijing, China) was used to measure the skeletal muscle mass. The instrument has five frequencies (5, 50, 100, 250, and 500 kHz) and is considered to be minimally affected by fluid overload when it is used to estimate muscle mass. The measurement of body mass by BCA-2A has been published elsewhere (10). The appendicular skeletal muscle mass (ASM) was calculated as the sum of lean muscle mass in the arms and legs.

Appendicular Skeletal Muscle Mass Index (ASMI) was calculated as the ratio of ASM to the square of height (ASM/height^2^).

Gait speed (GS) was used to assess physical performance. The subject was asked to walk 4 meters at a normal speed, and the time from the beginning of walk to the terminal point was recorded, followed by the calculation of GS.

### Definition of Sarcopenia

Sarcopenia was defined according to the updated EWGSOP2 consensus. First, the SARC-F scale was used to identify the subjects with sarcopenia. Then, handgrip strength was determined, the muscle mass was measured with BIA, and the severity of sarcopenia was assessed by GS assessment. Low handgrip strength was classified as possible sarcopenia, and sarcopenia was diagnosed based on low handgrip strength and low muscle quantity. Severe sarcopenia was defined as low handgrip strength, low muscle quantity, and low physical performance. The cut-off values are shown in Table 1.

**Table 1:** Diagnostic criteria for sarcopenia.

	**Male**	**Female**
[1] Low HS	<27 kg	<16 kg
[2] Low ASMI	<7.0 kg/m^2^	<6.0 kg/m^2^
[3] Low GS	≤ 0.8 m/s	
Diagnostic criteria	possible sarcopenia: (1); sarcopenia: (1) + (2); severe sarcopenia: (1) + (2) + (3)	

*Probable sarcopenia is identified by Criterion 1; Diagnosis is confirmed by additional documentation of Criterion 2; If Criteria 1, 2, and 3 are all met, severe sarcopenia is considered. HS: handgrip strength; ASMI: appendicular skeletal muscle mass index; GS: gait speed*.

### Statistical Analysis

Statistical analysis was performed with SPSS version 20.0 (IBM). The quantitative data with normal distribution are expressed as mean ± standard deviation and compared with analysis of variance among groups, followed by Bonferroni test for paired comparison. The quantitative data with abnormal distribution are expressed as median (Q_25_, Q_75_) and compared with Kruskal-Wallis rank sum test among groups, followed by the DSCF test. Multivariable analysis was performed by using modified Poisson regression analysis with robust sandwich variance to obtain the adjusted prevalence ratios (aPR) of low handgrip strength and potential confounding variables. First, univariate analysis was employed to determine associations between low handgrip strength and other variables. The Chi-square test, Mann-Whitney *U* test, and Wilcoxon rank-sum test were employed for the analysis of categorical, continuous, and ordinal variables, respectively. All independent variables with *P* < 0.1 in the univariate analysis were further included for multivariable regression analysis.

## Results

### General Characteristics

A total of 572 disabled subjects who met the inclusion criteria were included for final analysis. Visual disability, hearing disability, physical disability, and other types of disability accounted for 18.5, 8.4, 71.0, and 2.1%, respectively (multiple disabilities in nine subjects; speech disability in one, intellectual disability in one, and psychical disability in one). All these subjects received a questionnaire survey and measurements with the help of relatives or care providers. Level 4 disability had the highest proportion (68.4%), followed by level 3 disability (23.1%), and severe disability accounted for 8.5% (level 1 and 2).

Among these subjects, the mean age was 66.2 ± 4.2 years (range: 60–80 years). The male to female ratio was 1:1107. The mean body mass index (BMI) was 24.9 ± 3.6 kg/m^2^. According to the Chinese BMI-based criteria, subjects with normal BMI accounted for 47.2%, subjects who were overweight (BMI: 24.0–27.9) for 37.6%, and subjects with obesity (≥28.0) for 11.4% and those being lean (<18.5) for 3.8%. 79.7% of the subjects had at least one kind of chronic disease, such as hypertension (47.6%), heart diseases (23.4%), and type 2 DM (17.3%). The MNA-SF scale was used to screen for malnutrition. There are six questions in the MNA-SF scale, and the total score is 14. Score 12–14 is suggestive of normal nutritional status; score 8–11 is suggestive of risk or possibility for malnutrition; score 0–7 is suggestive of malnutrition. Results showed 14.3% of subjects had a risk of malnutrition, and 0.9% had malnutrition. The Chinese version of the PASE scale was employed to assess physical performance in the elderly. There are 32 items in the PASE scale which evaluate the physical activities (housework and leisure activities) in the past week. The total weighted score of 12 items ranges from 0 to 360, and the higher the score, the more physical activities are carried out (11). The median score of PASE scale was 78.57. There was no significant difference in the PASE among subjects with different types of disability. The remaining demographic characteristics are shown in Table 2.

**Table 2:** Demographic characteristics of subjects recruited.

**Variables**	**Total (*n* = 572)**	**Type of disability**				***Statistics***	***P***
		**Vision (*n* = 106)**	**Hearing (*n* = 48)**	**Physical (*n* = 406)**	**Others (*n* = 12)**		
Gender, *n* (%)							
M	301 (52.6)	54 (50.9)	24 (50.0)	215 (53.0)	8 (66.7)	1.220	0.748
F	271 (47.4)	52 (49.1)	24 (50.0)	191 (47.0)	4 (33.3)		
Age (y) $\bar{X}\pm SD$	66.2 ± 4.2	66.5 ± 0.4	68.2 ± 5.5	65.8 ± 3.9	67.4 ± 3.8	5.373	0.001^a^
Degree of disability, *n* (%)							
1 and 2	49 (8.6)	22 (20.8)	9 (18.8)	14 (3.4)	4 (33.3)		<0.001^b^
3	132 (23.1)	11 (10.4)	14 (29.2)	102 (25.1)	5 (41.7)		
4	391 (68.4)	73 (68.9)	25 (52.1)	290 (71.4)	3 (25.0)		
Education level *n* (%)							
Primary school or lower	33 (5.8)	8 (7.5)	4 (8.3)	21 (5.2)	0 (0.0)	8.879	0.449
Junior high school	306 (53.5)	52 (49.1)	21 (43.8)	226 (55.7)	7 (58.3)		
Senior school/technical secondary school/technical school	169 (29.5)	33 (31.1)	19 (39.6)	115 (28.3)	2 (16.7)		
College or higher	64 (11.2)	13 (12.3)	4 (8.3)	44 (10.8)	3 (25.0)		
Empty nest elderly *n* (%)							
Yes	355 (62.1)	63 (59.4)	31 (64.6)	252 (62.1)	9 (75.0)	1.294	0.731
No	217 (37.9)	43 (40.6)	17 (35.4)	154 (37.9)	3 (25.0)		
Income (Yuan/month) ^c^ M(*Q_25_, Q_75_*)	3500 (2333,4000)	3500 (3000,4000)	3500 (2833,4000)	3333 (2305,4000)	3625 (2750,3887)	3.219	0.359
Number of chronic diseases *n* (%)							
No	116 (20.3)	23 (21.7)	11 (22.9)	80 (19.7)	2 (16.7)	13.263	0.152
1	191 (33.4)	34 (32.1)	21 (43.8)	133 (32.8)	3 (25.0)		
2	144 (25.2)	32 (30.2)	10 (20.8)	101 (24.9)	1 (8.3)		
3 or more	121 (21.2)	17 (16.0)	6 (12.5)	92 (22.7)	6 (50.0)		
BMI (kg/m^2^) $\bar{X}\pm SD$	24.9 ± 3.6	24.8 ± 3.4	24.5 ± 3.5	24.9 ± 3.6	26.9 ± 4.6	1.529	0.206
Smoking status *n* (%)							
Smoker	388 (68.1)	78 (74.3)	41 (85.4)	261 (64.4)	8 (66.7)	11.998	0.007
Non-smoker	182 (31.9)	27 (25.7)	7 (14.6)	144 (35.6)	4 (33.3)		
Drinking *n* (%)							
No	431 (75.5)	83 (79.0)	42 (87.5)	300 (73.9)	6 (50.0)	9.233	0.026
Yes	140 (24.5)	22 (21.0)	6 (12.5)	106 (26.1)	6 (50.0)		
Depression *n* (%)							
Yes	459 (80.4)	89 (84.8)	43 (89.6)	317 (78.1)	10 (83.3)	5.287	0.125
No	112 (19.6)	16 (15.2)	5 (10.4)	89 (21.9)	2 (16.7)		
Fall in past year *n* (%)							
No	458 (80.1)	88 (83.0)	40 (83.3)	321 (79.1)	9 (75.0)	1.372	0.712
Yes	114 (19.9)	18 (17.0)	8 (16.7)	85 (20.9)	3 (25.0)		
MNA-SF score *n* (%)							
Normal	485 (84.8)	95 (89.6)	41 (85.4)	341 (84.0)	8 (66.7)	5.192	0.159
Risk for malnutrition	82 (14.3)	11 (10.4)	7 (14.6)	60 (14.8)	4 (33.3)		
Malnutrition	5 (0.9)	0 (0.0)	0 (0.0)	5 (1.2)	0 (0.0)		
PASE score, M(*Q_25_, Q_75_*)	78.57 (50.71,105.71)	78.57 (58.21,105.71)	79.29 (51.07,107.14)	78.57 (50.54,105.14)	84.66 (56.43,110.54)	2.501	0.475
SARC-F score, n(%)							
Normal	486 (85.0)	100 (94.3)	44 (91.7)	332 (81.8)	10 (83.3)	14.261	0.003
Risk for sarcopenia	86 (15.0)	6 (5.7)	4 (8.3)	74 (18.2)	2 (16.7)		

a *mean difference of hearing disability vs. physical disability (Bonferroni test) was 2.4 years (95% CI: 0.7-4.1), P < 0.001*;

b *Fisher exact test*;

c *Data available in 364 subjects and data unavailable in 208 subjects*.

### Prevalence of Sarcopenia

#### Screening for Sarcopenia

In this study, the Chinese edition of the SARC-F scale was employed to screen sarcopenia in 572 disabled elderly in Chinese communities. Results showed 15.0% (95% CI: 12.2–18.2%) of subjects had risk for sarcopenia, and there was significant difference in the proportion of subjects with risk for sarcopenia among subjects with different types of disability (χ^2^= 14.261, *P* = 0.003). In addition, the proportion of subjects with risk for sarcopenia in the physical disability group was higher than in the visual disability group (the difference between proportions was 12.6%, 95% CI: 8.7–16.5%; *P* = 0.002).

#### Assessment and Diagnosis of Sarcopenia

**Figure 2:**
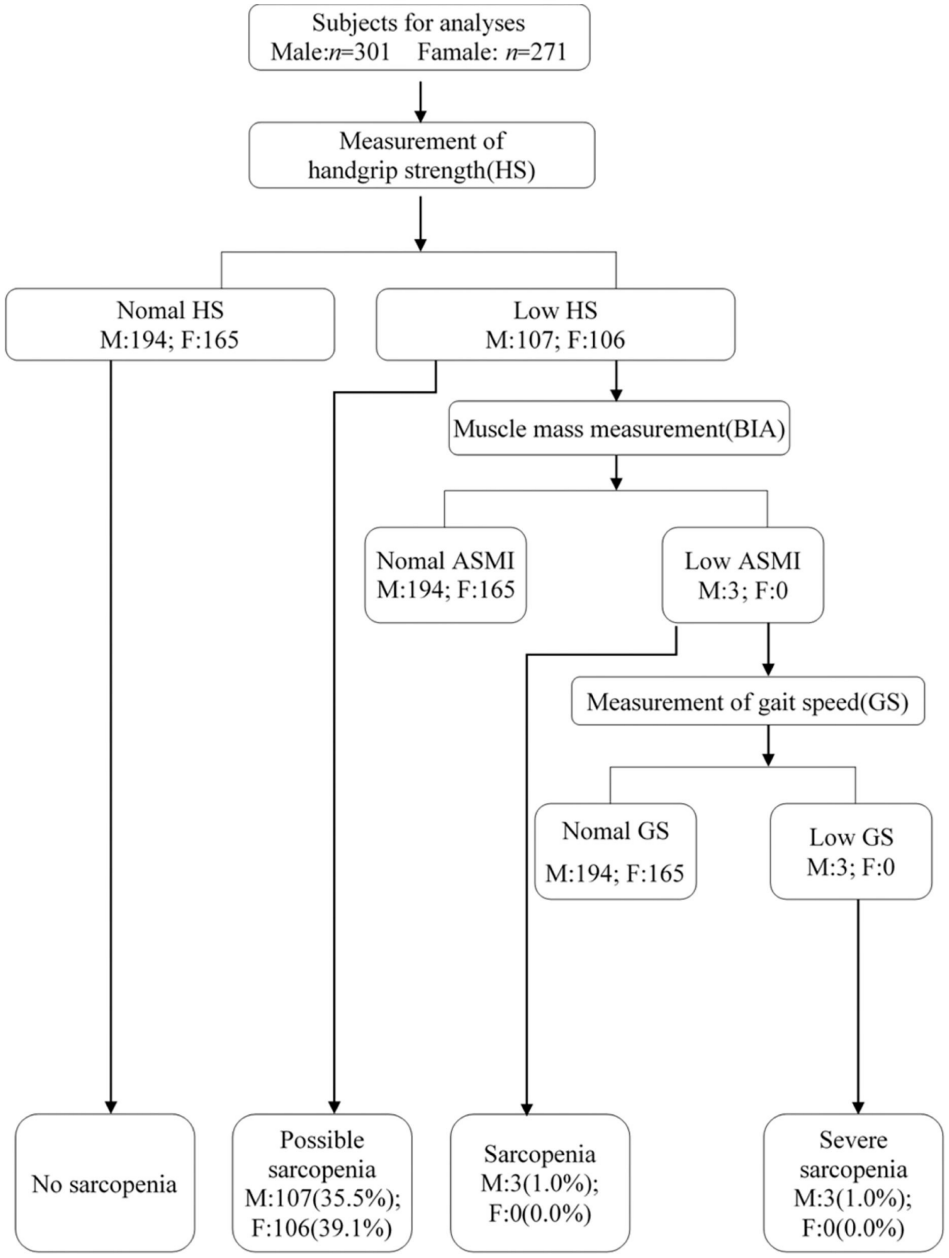
Study profile using the EWGSOP2019 criteria.

According to the EWGSOP2 consensus, the prevalence of possible sarcopenia was 37.2% (95% CI: 33.3–41.3%), the prevalence of sarcopenia was 0.5% (1.0% for male), and the prevalence of severe sarcopenia was 0.5% (Figure 2).

Table 3 displays the HS, ASMI, and GS of 572 subjects in the present study. The GS was 30.2 ± 9.0 kg (95% CI: 29.1-31.2) in males and 17.9 ± 6.0 kg (95% CI: 17.2-18.6) in females. The ASMI was 8.7 ± 0.9 kg/m^2^ (95% CI: 8.6-8.8) in males and 7.7 ± 0.8 kg/m^2^ (95% CI: 7.6-7.8) in females.

**Table 3:** HS, ASMI, and GS in different genders, types of disability, and age groups.

**Variables**	**HS (kg)**		**ASMI (kg/m**^****2****^**)**		**GS (m/s)**	
	**M**	***F***	**M**	***F***	**M**	***F***
Types of disability						
Visual	31.9 ± 8.2	19.5 ± 6.2	8.7 ± 0.9	7.7 ± 0.8	0.9 (0.8,0.9)	0.9 (0.8,0.9)
Hearing	28.6 ± 8.4	19.9 ± 5.8	8.6 ± 0.9	7.7 ± 0.8	0.9 (0.8,1.0)	0.9 (0.8,0.9)
Physical	30.3 ± 9.4	17.3 ± 5.9	8.7 ± 0.9	7.7 ± 0.8	0.9 (0.8,0.9)	0.9 (0.8,0.9)
Others	29.1 ± 6.6	15.5 ± 2.6	8.8 ± 1.3	8.3 ± 0.9	1.0 (0.9,1.0)	0.9 (0.9,0.9)
Age group						
60-64 years	32.5 ± 8.7	18.0 ± 6.5	8.7 ± 0.9	7.6 ± 0.8	0.9 (0.8,0.9)	0.9 (0.8,1.0)
65-69 years	29.0 ± 9.2	18.1 ± 5.7	8.6 ± 0.9	7.8 ± 0.8	0.9 (0.8,1.0)	0.9 (0.8,0.9)
70-74 years	28.5 ± 8.9	17.1 ± 5.4	8.7 ± 0.8	7.7 ± 0.8	0.9 (0.8,0.9)	0.9 (0.8,0.9)
≥75 years	26.5 ± 7.8	19.1 ± 5.6	8.6 ± 1.0	8.1 ± 0.8	0.8 (0.8,1.0)	1.0 (0.8,1.1)
Total	30.2 ± 9.0	17.9 ± 6.0	8.7 ± 0.9	7.7 ± 0.8	0.9 (0.8,0.9)	0.9 (0.8,0.9)

#### Factors Associated With Low Muscle Strength

The modified Poisson regression model (adjusted PR and corresponding 95% CI) was used to evaluate the relationship among related variables and low handgrip strength because of the relatively high prevalence of low handgrip strength (12) (Table 4).

**Table 4:** Social-demographics characteristics, types of disability, depression, MNA-SF score, PASE score, and low handgrip strength.

**Variables**	**Low handgrip strength**				**Crude PR**	**95% *CI***	**Adjusted PR**	**95% *CI***
	**Total (*n* = 572)**	**Yes (*****n*** **=** **572)**		***P***				
		***n***	**%**					
Gender								
Male	301	107	35.5	0.378	1		1	
Female	271	106	39.1		1.10	0.89–1.36	1.04	0.85–1.29
Age group								
60-64 years	229	76	33.2	0.078^a^	1		1	
65-69 years	220	85	38.6		1.16	0.91–1.49	1.08	0.85–1.37
70-74 years	103	43	41.7		1.26	0.94–1.69	1.19	0.90–1.57
≥75 years	20	9	55.0		1.36	0.81–2.28	1.35	0.80–2.31
Types of disability							
Vision	106	24	22.6	0.001^b^	1		1	
Hearing	48	14	29.2		1.29	0.73–2.26	1.23	0.70–2.17
Physical	406	168	41.4		1.83	1.26–2.65	1.69	1.18–2.42
Others	12	7	58.3		2.58	1.42–4.67	2.45	1.34–4.49
Depression								
No	459	150	32.7	<0.001	1		1	
Yes	112	63	56.2		1.72	1.40–2.12	1.31	1.04–1.62
MNA-SF								
Normal	485	166	34.2	<0.001	1		1	
Risk for malnutrition and malnutrition	87	47	54.0		1.58	1.25–1.99	1.27	0.99–1.62
PASE score, M(*Q_25_, Q_75_*)	78.57 (50.71,105.71)	62.14(33.57,93.57)	-	<0.001^c^	0.99	0.99–0.99	0.99	0.99–1.00

a *Chi-square test*;

b *Fisher exact test*;

c *Mann-Whitney U test*.

Our results showed the types of disability, depression, and PASE score were associated with low HS. In the physically disabled elderly, the risk for low HS was 1.69 times (aPR: 1.69, 95% CI: 1.88-2.42) that in the elderly with visual disabilities. This risk for low HS in the elderly with depressive symptoms was 31% (aPR: 1.31, 95% CI: 1.04-1.62) higher than in those without depressive symptoms. PASE score was a protective factor for HS (aPR: 0.99, 95% CI: 0.99-1.00). In the univariate analysis, significant association (cPR: 1.58, 95% CI: 1.25-1.99) was observed between low handgrip strength and MNA-SF score. However, this association was not observed in the adjusted analysis (aPR: 1.27, 95% CI: 0.99-1.62).

## Discussion

The prevalence of sarcopenia in the elderly varies widely among countries. A systematic review reveals that the prevalence of sarcopenia is 1–29% among elderly people in the general public, 14–33% among residents in nursing facilities, and 10% in hospitalized patients based on the diagnostic criteria of the EWGSOP (2010) consensus (13). In Asian countries, the prevalence of sarcoma is between 5.5 and 25.7% (5.1–21.0% for men and 4.1–16.3% for women) based on the diagnostic criteria of AWGS (2014) consensus. Our previous review indicated that the prevalence of sarcopenia in China (14) was 1.3–23.6% for men and 0.3–21.6% for women.

This study investigated for the first time the prevalence of sarcopenia among the disabled elderly in a Chinese community, and the updated EWGSOP consensus was used. In this study, the prevalence of sarcopenia was only 1.0% in men. Some studies on sarcopenia have also reported a low prevalence of sarcopenia (4, 15). Although a higher prevalence of sarcopenia has also been reported in China, the included subjects were largely older than 70 years. However, in the present study, the age of subjects ranged from 60 years to 70 years and the median age was 66 years (Table 4). This indicates that older subjects are susceptible to sarcopenia.

The prevalence of low muscle strength was high, which was consistent with some epidemiological studies in Asia (16, 17). There is growing evidence showing that muscle strength and function decline to a greater extent than muscle mass (15, 18). In addition, muscle strength or performance is more closely associated with disability, morbidity, and mortality as compared to muscle mass (19). This suggests that the reduction of muscle strength is a key feature of sarcopenia, which is also in line with the updated EWGSOP consensus on sarcopenia in 2018. The updated AWGS consensus in 2019 also recommends the identification of people with “possible sarcopenia” based on decreased muscle strength and / or physical performance (2). According to the definition of low muscle strength (HS: <28 kg for male, <18 kg for female) in this new consensus, the proportion of disabled subjects with low handgrip strength will be higher in this study.

The disabled elderly with physical disabilities seemed to be more likely to be at risk for low HS than those with a visual disability. People with a physical disability have difficulties in limb movement, and thus it is reasonable to expect a decline in HS. A physical disability itself can lead to reduced skeletal muscle mass and reduced stimulation to skeletal muscles, which in turn cause significant muscular atrophy over time. Without intervention, it will cause a vicious cycle.

Our result indicated no correlation between health status of the elderly and reduced handgrip strength. However, it has been reported that chronic diseases might cause reduced muscular strength, and also some drugs for treatment of chronic diseases would influence muscular strength (20).

Our study showed that the disabled elderly with depressive symptoms based on the Geriatric Depression Rating Scale had a higher risk for low HS than those without depressive symptoms. Studies on the elderly in the general population have also shown a relationship between HS and depression (21). Based on the findings from the 2011–2014 National Health and Nutrition Survey, Brooks et al. (22) found that HS was negatively related to depression among those over 60 years in the communities. A recent study on the relationship between sarcopenia and depressive symptoms in the elderly (23) revealed that, after adjustment for age, gender, and other potential confounding factors, reduced muscle mass was closely associated with depressive symptoms (OR: 2.23, 95% CI 1.06-4.92). Available studies have shown that the detection rate of depressive symptoms is relatively high among the disabled elderly (24, 25). However, available studies, including the present study, are cross-sectional, and more cohort studies are needed to investigate the causal relationship between low HS/sarcopenia and depressive symptoms. As compared to the non-disabled elderly, the PASE score was lower in the disabled elderly (11, 26), which is understandable. However, daily physical activity is still a protective factor for HS. Thus, it is necessary for the disabled elderly to train the upper limbs as much as possible.

There were still limitations in the present study. The disabled elderly who could not receive BIA were excluded from this study, such as subjects with severe hemiplegia or implantation of prostheses and metal plates. Moreover, HS and GS were measured in our study, and thus some people with poor health status might not have participated in our study. This will affect the real prevalence of sarcopenia. Also, subjects with intellectual disabilities or psychiatric disabilities were not included in our study, and these people are often excluded from studies. However, they are also disabled people and account for 14% of the disabled people in China. Several studies have shown that they do not receive enough exercise and therefore have a higher risk for malnutrition-related diseases (such as obesity and cancer) (27). Thus, more attention should be paid to the reduction of muscle mass in people with intellectual or mental disabilities. Finally, in the present study, the data about daily dietary energy and specific nutrients (such as vitamin D, vitamin B12, etc.) were not recorded. However, these play a key role in the occurrence and development of sarcopenia (28, 29).

## Conclusion

In summary, the prevalence of EWGSOP2-defined sarcopenia is low and the prevalence of declined muscle strength is high in a community of Shanghai. The elderly with a physical disability had a higher prevalence of low HS than those with a visual disability. In the future, more studies with a large sample size and long-term follow-up are needed to confirm our findings in the disabled elderly.

## Data Availability Statement

The raw data supporting the conclusions of this article will be made available by the authors, without undue reservation.

## Ethics Statement

The studies involving human participants were reviewed and approved by Ethics Committee of Putuo People's Hospital, Shanghai. The patients/participants provided their written informed consent to participate in this study.

## Author Contributions

QF and GZ designed the work, acquired and analyzed data, and participated in writing the manuscript. JH, SP, MF, QL, QY, XL, and QT acquired and analyzed data and revised the manuscript. DH and JL contributed to the concept and design of the work and reviewed and revised the manuscript. All authors contributed to the article and approved the submitted version.

## Conflict of Interest

The authors declare that the research was conducted in the absence of any commercial or financial relationships that could be construed as a potential conflict of interest.

## References

[1] Cruz-Jentoft AJ Bahat G Bauer J Boirie Y Bruyere O Cederholm T Sarcopenia: revised European consensus on definition and diagnosis. Age Ageing. (2019) 48:16–31. 10.1093/ageing/afy169 30312372 PMC6322506

[2] Chen LK Woo J Assantachai P Auyeung TW Chou MY Iijima K. Asian working group for sarcopenia: 2019 consensus update on sarcopenia diagnosis and treatment. J Am Med Dir Assoc. (2020) 21:300–7 e302. 10.1016/j.jamda.2019.12.012 32033882

[3] Chen M Bai HJ Wang C Wang Y Xv DF Xie H Establishment of muscle mass diagnostic standard of sarcopenia using a bioelectrical impedance analysis and epidemiological investigation of the elderly in Shanghai (in Chinese). Chin J Geriatrics. (2015) 34:483–6. 10.3760/cma.j.issn.0254-9026.2015.05.007

[4] Zeng P Wu SN Han YW Gong H Pang J Zhang Y Epidemiologic features of sarcopenia and its related indexes in elderly population in Beijing. Chin J. Geriatrics. (2015) 34:478–82.

[5] Malmstrom TK Morley JE SARC-F: a simple questionnaire to rapidly diagnose sarcopenia. J Am Med Dir Assoc. (2013) 14:531–2. 10.1016/j.jamda.2013.05.018 23810110

[6] Malmstrom TK Miller DK Simonsick EM Ferrucci L Morley JE. SARC-F: a symptom score to predict persons with sarcopenia at risk for poor functional outcomes. J Cachexia Sarcopenia Muscle. (2016) 7:28–36. 10.1002/jcsm.12048 27066316 PMC4799853

[7] Woo J Leung J Morley JE. Validating the SARC-F: a suitable community screening tool for sarcopenia? J Am Med Dir Assoc. (2014) 15:630–4. 10.1016/j.jamda.2014.04.021 24947762

[8] Yang M Hu X Xie L Zhang L Zhou J Lin J. Comparing mini sarcopenia risk assessment with SARC-F for screening sarcopenia in community-dwelling older adults. J Am Med Dir Assoc. (2019) 20:53–7. 10.1016/j.jamda.2018.04.012 29909052

[9] Yang M Lu J Jiang J Zeng Y Tang H. Comparison of four sarcopenia screening tools in nursing home residents. Aging Clin Exp Res. (2019) 31:1481–9. 10.1007/s40520-018-1083-x 30539542

[10] Zeng P Wu S Han Y Liu J Zhang Y Zhang E. Differences in body composition and physical functions associated with sarcopenia in Chinese elderly: reference values and prevalence. Arch Gerontol Geriatr. (2015) 60:118–23. 10.1016/j.archger.2014.08.010 25440136

[11] Vaughan K Miller WC. Validity and reliability of the Chinese translation of the Physical Activity Scale for the Elderly (PASE). Disabil Rehabil. (2013) 35:191–7. 10.3109/09638288.2012.690498 22671717 PMC3540101

[12] Zou GY Donner A. Extension of the modified Poisson regression model to prospective studies with correlated binary data. Stat Methods Med Res. (2013) 22:661–70. 10.1177/0962280211427759 22072596

[13] Cruz-Jentoft AJ Landi F Schneider SM Zuniga C Arai H Boirie Y. Prevalence of and interventions for sarcopenia in ageing adults: a systematic review. Report of the International Sarcopenia Initiative (EWGSOP and IWGS). Age Ageing. (2014) 43:748–59. 10.1093/ageing/afu115 25241753 PMC4204661

[14] Fang Q Zhu G Fang M Huang D Progress on the screening of sarcopenia and the prevalence in chinese elderly (in Chinese). Health Educ Health Prom. (2018) 13:526–9.

[15] Zeng P Han Y Pang J Wu S Gong H Zhu J. Sarcopenia-related features and factors associated with lower muscle strength and physical performance in older Chinese: a cross sectional study. BMC Geriatr. (2016) 16:45. 10.1186/s12877-016-0220-7 26879964 PMC4754915

[16] Shimokata H Ando F Yuki A Otsuka R. Age-related changes in skeletal muscle mass among community-dwelling Japanese: a 12-year longitudinal study. Geriatr Gerontol Int. (2014) 14 Suppl 1:85–92. 10.1111/ggi.12219 24450565

[17] Kim YH Kim KI Paik NJ Kim KW Jang HC Lim JY. Muscle strength: a better index of low physical performance than muscle mass in older adults. Geriatr Gerontol Int. (2016) 16:577–85. 10.1111/ggi.12514 26017097

[18] Xu HQ Shi JP Shen C Liu Y Liu JM Zheng XY. Sarcopenia-related features and factors associated with low muscle mass, weak muscle strength, and reduced function in Chinese rural residents: a cross-sectional study. Arch Osteoporos. (2018) 14:2. 10.1007/s11657-018-0545-2 30560296

[19] Meng NH Li CI Liu CS Lin CH Lin WY Chang CK. Comparison of height- and weight-adjusted sarcopenia in a Taiwanese metropolitan older population. Geriatr Gerontol Int. (2015) 15:45–53. 10.1111/ggi.12227 24397819

[20] Rizzo MR Barbieri M Fava I Desiderio M Coppola C Marfella R. Sarcopenia in elderly diabetic patients: role of dipeptidyl peptidase 4 inhibitors. J Am Med Dir Assoc. (2016) 17:896–901. 10.1016/j.jamda.2016.04.016 27262494

[21] Stessman J Rottenberg Y Fischer M Hammerman-Rozenberg A Jacobs JM. Handgrip strength in old and very old adults: mood, cognition, function, and mortality. J Am Geriatr Soc. (2017) 65:526–32. 10.1111/jgs.14509 28102890

[22] Brooks JM Titus AJ Bruce ML Orzechowski NM Mackenzie TA Bartels SJ. Depression and handgrip strength among, U.S. adults aged 60 years and older from NHANES 2011-2014. J Nutr Health Aging. (2018) 22:938–43. 10.1007/s12603-018-1041-5 30272097 PMC6168750

[23] Zhang Y Hao Q Ge M Dong B. Association of sarcopenia and fractures in community-dwelling older adults: a systematic review and meta-analysis of cohort studies. Osteoporos Int. (2018) 29:1253–62. 10.1007/s00198-018-4429-5 29500527

[24] Yang Z Liu Y Xue L Ren X Depressive symptoms and its related factors among the physical disabled aged 45 years or older: the data from 2013 China Health and Retirement Longitudinal Study (in Chinese). Chinese Mental Health J. (2017) 31:585–9.

[25] Lee K So WY. Differences in the Levels of Physical Activity, Mental Health, and Quality of Life of Elderly Koreans with Activity-Limiting Disabilities. Int J Environ Res Public Health. (2019) 16:15. 10.3390/ijerph16152736 31370296 PMC6696124

[26] Hagiwara A Ito N Sawai K Kazuma K. Validity and reliability of the Physical Activity Scale for the Elderly (PASE) in Japanese elderly people. Geriatr Gerontol Int. (2008) 8:143–51. 10.1111/j.1447-0594.2008.00463.x 18821997

[27] Koritsas S Iacono T. Weight, nutrition, food choice, and physical activity in adults with intellectual disability. J Intellect Disabil Res. (2016) 60:355–64. 10.1111/jir.12254 26712472

[28] An R Chiu CY Zhang Z Burd NA. Nutrient intake among US adults with disabilities. J Hum Nutr Diet. (2015) 28:465–75. 10.1111/jhn.12274 25233949

[29] Yang LJ Wu GH Yang YL Wu YH Zhang L Wang MH. Nutrition, physical exercise, and the prevalence of sarcopenia in elderly residents in nursing homes in China. Med Sci Monit. (2019) 25:4390–9. 10.12659/MSM.914031 31189870 PMC6587647

